# Electrode Modified by Reduced Graphene Oxide for Monitoring of Total Thallium in Grain Products

**DOI:** 10.3390/ijerph15040653

**Published:** 2018-04-01

**Authors:** Bozena Karbowska, Tomasz Rębiś, Grzegorz Milczarek

**Affiliations:** Institute of Chemistry and Technical Electrochemistry, Poznan University of Technology, Berdychowo 4, Poznan 61-965, Poland; tomasz.rebis@put.poznan.pl (T.R.); grzegorz.milczarek@put.poznan.pl (G.M.)

**Keywords:** thallium, grain products, reduced graphene oxide, differential-pulse anodic stripping voltammetry

## Abstract

Grain products and the associated industry have a notable economic and social impact all over the world. The toxicological safety of grain products is a nutritional prerogative. This study focused on the determination of thallium content in grain product samples collected from a commercial brand commonly available in Poland. The samples were analyzed with the use of differential pulse anodic stripping voltammetry (DPASV) with graphene oxide based on glassy carbon. The stripping anodic peak current of thallium was linear over its concentration range from 9.78 × 10^−9^ to 97.8 × 10^−9^ M. The limit of detection (LOD) was calculated according to the formula LOD = (κ × SDa)/b, where κ is 3.3, SDa is the standard deviation of the intercept, and b is the slope. The determined value of LOD was 1.229 µg L^−1^ (6.01 × 10^−9^ M). The proposed method was successfully applied for the determination of thallium ions in samples of actual grain products. The obtained results confirmed that thallium was present in the studied cereal samples (average content at 0.0268 ± 0.0798 mg/kg). Thallium has a half-life of 60 days; therefore, the consumption of foods with thallium content of approximately 0.08 mg/kg has the potential for harmful bioaccumulation in the body. Thallium contamination in cereal products should be a critical parameter for health environmental regulations.

## 1. Introduction

Flour and grain products are an important source of nutrients and, as such, are widely consumed in the majority of the world. Since flour is a commodity in the bread and bakery industry, its safety is of high nutritional and toxicological interest. Due to the high consumption rate of flour-based products, the presence of hazardous heavy metal, such as thallium (Tl), would be a significant health risk. Tl(III) is approximately 1000 times more toxic compared with Tl(I) [1]. The presence of contaminants comprising thallium in air results in its absorption by plants—a process that is further enhanced by high thallium content in soil [2,3]. Samples of clovers collected from uncontaminated regions of Poland contained 0.008–0.01 mg/kg thallium. In the case of grasses, concentrations ranged from 0.02 to 0.6 mg/kg, and these values reached 0.02–0.3 mg/kg for vegetables, whereas fungi contained up to 5.5 mg/kg [4,5]. Although there is little information regarding this subject, breads, cereals, pasta, and flour can also contain thallium (Tl). Due to its similarity to alkali metals, it can replace potassium in biological systems. Tl+ is known to interfere with Na^+^/K^+^ ATPase and pyruvate kinase [6].

The selection of an appropriate analytical technique for quantitative and qualitative determination of thallium depends on the physical state of the sample, its volume, possible decomposition, and processing options. However, the sensitivity, detection and quantification ranges, selectivity, and precision should be treated as the main criteria.

Anodic stripping voltammetry (ASV) has long been recognized as a powerful technique for determination of trace metals (e.g., zinc, lead, copper, and thallium) due to its high sensitivity and relatively inexpensive instrumentation [7]. When measurements are conducted in the presence of complexing agents such as ethylenediaminetetraacetic acid (EDTA), ASV enables trace thallium determination in the presence of large amounts of interfering heavy metal ions. ASV allows for the determination of Tl(I) in plant samples or soil extracts with a limit of detection comparable to ICP MS [8]. Previously, several solid electrodes were evaluated for the determination of trace metals. According to the literature, noble metal electrodes, such as silver and gold [9,10], transition metal electrodes (bismuth film, antimony-based electrodes) [11,12], and various carbon electrodes [13,14,15] are the most representative examples. For instance, the reliable, accurate, and fast detection of thallium is of great significance due to the very high toxicity of this metal [3]. The electrochemical determination of thallium by ASV has the advantages of high sensitivity and good selectivity. The electrochemical determination of thallium using mercury electrodes has been the most extensively studied method [16,17]. Moreover, electrodes modified with nanomaterials supported on multi-walled carbon nanotubes [18] or a graphene/ionic liquid matrix have also been investigated for thallium determination [19]. 

In recent years, chemically and electrochemically reduced graphene oxide (RGO) has attracted great attention for the modification of electrode surfaces. This is due to its high surface area, high electronic transport, and excellent electrocatalytic activity [20,21,22].

In this work, the applicability of ASV with reduced graphene oxide (RGO) on a glassy carbon electrode (GC) was investigated in order to determine thallium in flour and grain products. Determination of thallium by differential pulse ASV with GC/RGO was found to be a feasible and attractive approach.

## 2. Materials and Methods

### 2.1. Cereal Materials

Five widely consumed commercial grain product samples were purchased from different outlets. Buckwheat groats were acquired from the company Konpack, Konin (Poland); oatmeal porridge from the company MELVIT, Olszewo-Borki (Poland); white rice from the company Tira dell Burma (Asia); spaghetti from the company FIRENZE, Rzeszow (Poland); rye bread from the company Pod Strzechą, Poznan (Poland). These samples were transported as purchased to the laboratory for immediate preliminary treatment and subsequent analysis. All samples were air-dried in an oven at 55 °C for 24 h and pulverized in an agate planetary mill to a grain size of <0.06 mm. The decomposition of food samples was carried out according to the previously described procedure [23]. A sample of the studied grain products (0.5 g) was placed in a Teflon beaker and digested by adding 65% nitric acid and 2.5 mL of 30% hydrogen peroxide. Upon evaporation of the solution, the residues were mixed with an additional portion of nitric acid (1 mL), covered with a watch glass and heated for 3 h. After filtration, the residues were then mixed with ascorbic acid (2.5 mL of 1 M solution) and EDTA (6.25 mL of 0.2 M solution). The pH of the solution was then adjusted to a value of 4.5 (using an ammonium solution), then it was transferred to a flack (25 mL) and supplemented with water. 

### 2.2. Instrumentation

A µAutolab electrochemical analyzer from EcoChemie (Utrecht, Netherlands) was used for electrochemical measurements. A standard three-electrode configuration consisting of the glassy carbon modified working (GC, A = 0.07 cm^3^), Ag/AgCl (3 M KCl) reference and Pt wire counter electrodes was incorporated into a glass cell (V = 20 cm^3^). Scanning electron microscopy (SEM) was carried out using a HITACHI S-3400N microscope.

### 2.3. Chemicals and Reagents

A certified reference material—GBW 07401 soil of Chinese origin, containing 1 ± 0.2 μg/g of Tl—was used. Ammonia solution (25%), nitric acid (65%), hydrogen peroxide (30%), EDTA, and ascorbic acid (all puriss. p.a., provided by FLUKA) as well as hydrofluoric acid (pract. 73%, FLUKA) were used. Graphene oxide (4 mg/mL) was provided by Nanopoz (Poland). All solutions were prepared in water by reverse osmosis in a Watek-Demiwa 5 Rosa system (Czech Republic).

### 2.4. Preparation of Glassy Carbon/Reduced Graphene Oxide Modified Electrode (GC/RGO)

The preparation of the reduced graphene oxide modified electrode (GC/RGO) was realized as follows [21]. First, 1 µL of a GO solution (a 4 mg/ml water solution) was dropped on the surface of a GC electrode and dried in an oven at 60 °C to form a film. Then, electrochemical reduction of GO was conducted by cycling voltammetry in deoxygenated 0.05 M (pH 7.4) phosphate buffer (PB) by applying 10 cycles in the range between 0.4 and −0.9 V at 50 mV/s. 

The modified electrode prepared using the method described above can be re-used with no detectable decrease of sensitivity. However, we used one electrode for an entire day of electrochemical measurements. The regeneration of the electrode is possible and consists of two steps. First, the modifier layer is removed by polishing of the surface with Al_2_O_3_ water slurry (30–60 nm in diameter) on a polishing cloth. Next, a new portion of the same GO solution (the 4 mg/mL water solution) is placed on the surface of the pristine GCE and left to dry in the oven. Due to a very simple preparation step and fast regeneration, the electrode was regenerated after each set of thallium assays.

### 2.5. Thallium Determination by Differential Pulse Anodic Stripping Voltammetry (DPASV) at GC/RGO Electrode

After mineralization, the samples were analyzed with the use of differential pulse anodic stripping voltammetry (DPASV) on a glassy carbon electrode covered by reduced graphene oxide (GC/RGO). The conventional three electrode system was used in this investigation with GC/RGO as the working electrode, Pt wire as the counter electrode and Ag/AgCl (3 M KCl) as the reference electrode. EDTA (0.05 M) was used as the supporting electrolyte. Thallium pre-concentration was performed at a constant potential (chronoamperometry) of −1.2 V vs. Ag/AgCl and a deposition time of 600 s, a pulse amplitude of 50 mV, and a step potential of 2 mV. The applied accumulation potential is sufficiently low to reduce thallium ions to the metallic form. [24]. Control measurements were performed together with each series of experiments. Nine independent trials were conducted for the reference material (soil GBW 07401) in order to determine Tl content. The average content of Tl was at 0.90 ± 0.14 µg/g (with a minimum of 0.76 and a maximum of 1.1 µg/g).

## 3. Results and Discussion

Recently, reduced graphene oxide (RGO) has been considered as a perfect electrode material due to high conductivity, high surface area, and good electrocatalytic activity [20,21,22]. The electrochemical reduction of GO is an effective and environmentally friendly method for the formation of graphene-like conductive structures. This process can be simply monitored by cyclic voltammetry (CV), considering the fact that electroreduction of oxygen-containing surface groups of GO gives rise to a more conductive and roughly reduced graphene oxide (RGO) material.

**Figure 1:**
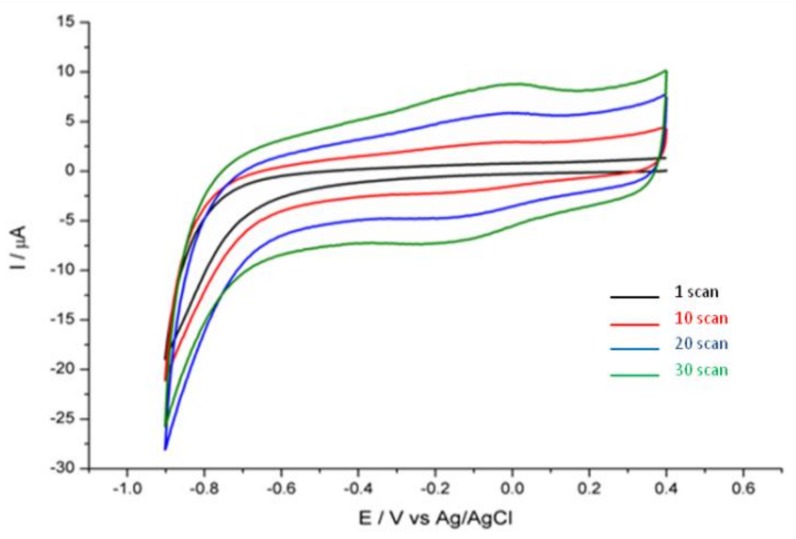
Cyclic voltammograms of the electrochemical reduction of graphene oxide at the glassy carbon (GC) electrode in 0.05 M phosphate buffer (PB) buffer (pH = 7.4) at 50 mV/s.

The electrochemical reduction of GO is shown in Figure 1. The CV was conducted in a deoxygenated PB buffer within a potential range from −0.9 to 0.4 V vs. Ag/AgCl. As can be observed in Figure 1, the high reduction current which appeared at approximately −0.8 V is a result of electrochemical reduction of surface oxygen functional groups from GO [20,25]. As the potential cycling proceeded, the redox peak currents at approximately −0.1 V increased and then the peak currents remained relatively constant after a certain number of scans, typically 15. The formation of similar peaks has been observed previously, when GO was reduced electrochemically [26].

**Figure 2:**
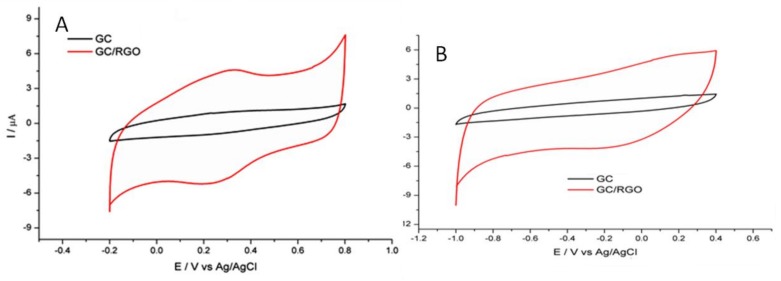
Cyclic voltammograms recorded for pristine GC and GC/reduce graphene oxide (RGO) in 0.1 M HClO_4_ (**A**) and 0.05 M ethylenediaminetetraacetic acid (EDTA) (**B**). Scan rate at 50 mV/s.

The comparison of post-electroreduction CVs in 0.1 M HClO_4_ and 0.05 M EDTA is presented in Figure 2A,B. It can be observed that the CVs of GC/RGO in both electrolytes exhibited notably higher capacitance compared to the pristine GC. The results suggest that the GC/RGO electrode possesses a significantly increased electroactive surface.

**Figure 3:**
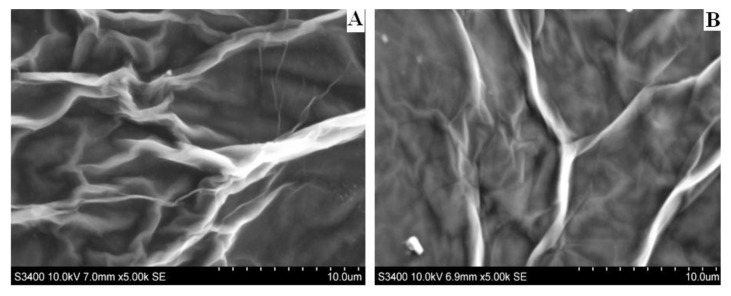
Scanning electron micrograph images of electrode surface—graphene oxide (**A**) and electrochemically reduced graphene oxide (**B**).

The morphology of GO and RGO materials was studied by scanning electron microscopy (SEM), as shown in Figure 3. GO sheets exhibited a crumpled and wrinkled thin sheet structure, which is in good agreement with other reports [20,21,26]. It can be observed in Figure 3B that the electrochemical reduction of the oxygen-based moieties derived from GO did not remarkably affect the morphology of the surface, but the RGO surface became slightly rougher.

### Determination of Thallium at GC/RGO Modified Electrode by DPASV

**Figure 4:**
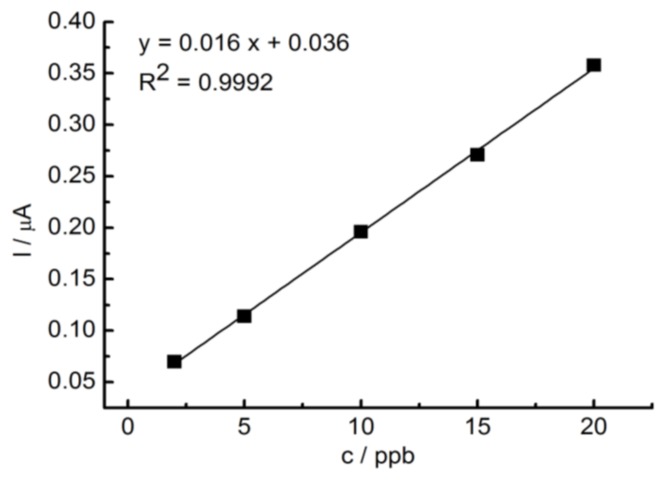
Calibration curve obtained for the GC/RGO electrode in EDTA after the addition of 2, 5, 10, 15, and 20 µg L^−1^ Tl^+^. Six hundred seconds of pre-concentration at −1.2 V vs. Ag/AgCl, a pulse amplitude of 50 mV, and a step potential of 2 mV.

During the successive additions of different amounts of Tl^+^ to 10 mL of EDTA, a well-defined response was obtained, indicating good electroanalytical properties of the modified electrode. A linear relationship between the anodic current and thallium concentration was observed over the range of 2–20 µg L^−1^, which corresponds to 9.78 × 10^−9^–97.8 × 10^−9^ M (Figure 4). The calibration curve was obtained by fitting the equation: y = 0.01x + 0.75 with a correlation coefficient (R2) of 0.9992. The limit of detection (LOD) was calculated according to the formula: LOD = (κ × SDa)/b, where κ is 3.3, SDa is the standard deviation of the intercept, and b is the slope. The determined value of LOD was 1.229 µg L^−1^ (6.01 × 10^−9^ M).

**Figure 5:**
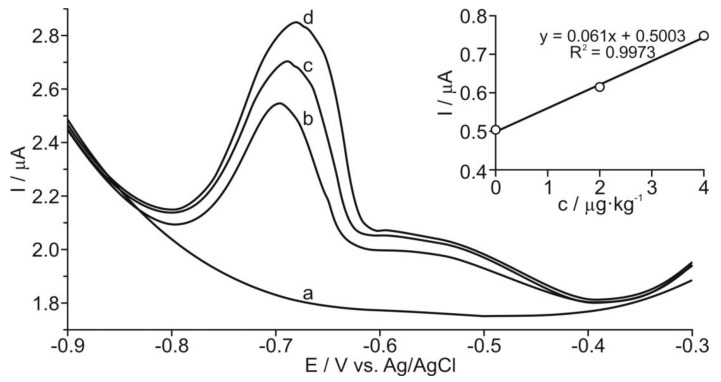
Voltammogram of the processed grain product sample (**b**) together with voltammograms of the sample with the sequential standard addition of (**c**) 2 ppb Tl and (**d**) 4 ppb Tl, as well as the calibration graph. (**a**) The base electrolyte: 0.05 M EDTA (pH = 4.5). Pre-concentration potential: −1.2 V vs. Ag/AgCl pre-concentration time: 600 s.

For the determination of thallium, the standard addition method previously described by Karbowska et al. [17] was adopted with slight modifications. Tl pre-concentration was carried out at a potential of –1.2 V vs. Ag/AgCl and deposition time of 600 s. Voltammograms of GC/RGO were recorded after medium exchange on pure 0.05 M EDTA. The differential-pulse amplitude was equal to 50 mV (Figure 4). The determination of peak height, which depended on the potential of Tl concentration, allowed for the selection of −1.2 V as the optimum potential for the study (Figure 5). The concentration of Tl in the samples was estimated on the basis of several standard additions (typically three additions). In order to determine the SD, each sample was measured three times (Figure 5, inset).

The obtained results confirmed that the employed DPASV method is effective in terms of the determination of thallium content in grain products. The results are presented in Table 1. 

Based on the results obtained in the framework of study, it can be concluded that thallium is present in cereal products in trace amounts ranging from 0.0268 to 0.0798 mg/kg. White rice is characterized by the lowest amount of thallium (only 0.0268 mg/kg), whereas the highest concentration of thallium (0.0798 mg/kg) was recorded in spaghetti noodles. There is a wide range of reported thallium concentrations in food plants, from 0.005 to 0.125 mg/kg. The dietary intake of thallium in the UK is estimated to be approximately 0.005 mg per day. In general, it is estimated that a daily diet contains 2 µg/kg thallium [27]. The average content of thallium in the human body measured in the U.S. population was approximately 0.1 mg [28].

In Europe, a maximum thallium level of 0.46 ± 2.24 mg/kg d.m. for animal forage has been proposed [29]. If the same maximum level of approximately 2 mg/kg d.m. of thallium is adopted for human food as a limit acceptable for human health, it is clear from the obtained data (Table 1) that buckwheat groats, oatmeal porridge, white rice, spaghetti, and rye bread would be safe for consumption. However, it should be noted that thallium has a half-life of 60 days, so the consumption of foods with thallium content of approximately 0.08 mg/kg has the potential for harmful bioaccumulation in the body. Very often there is no information regarding the limit values of thallium in cereal products. The toxicity of thallium is higher compared to mercury, cadmium, and lead (maximum admissible concentration at 0.1 mg/mL) [6,30]. The toxicity of thallium-based compounds is mainly caused by the similarity between thallium (I) ions and potassium ions [2,31].

## 4. Conclusions

An innovative and rarely used method of differential pulse anodic stripping voltammetry with graphene oxide based on glassy carbon was successfully used for the determination of thallium in grain products, and the obtained results allowed for a preliminary evaluation of Tl content in such products. Given the high intake of cereal products, a regular intake of food with high thallium content may be detrimental to health. It should also be emphasized that the effects of chronic exposure to low concentrations of thallium are currently unknown. The analytical data presented in this study may provide a basis for establishing the limits of thallium content in food, as the European Commission has been planning such actions with respect to lead and cadmium. Thallium contamination in cereal products should be considered a critical parameter, and health and environmental regulations should be amended accordingly.

## Figures and Tables

**Table 1:** Thallium concentration (mg/kg) in consumable commercial grain products.

Cereal Products	Arthmetic Mean (mg/kg)	Minimum (mg/kg)	Maximum (mg/kg)	Median (mg/kg)	Standard DeviationSD (mg/kg)
**Buctwheat groats**	0.0587	0.0449	0.0664	0.0648	0.0119
**Oatmeal porridge**	0.0334	0.0225	0.0419	0.0358	0.0099
**White rice**	0.0268	0.0205	0.0317	0.0282	0.0057
**Spagetti**	0.0798	0.0762	0.0845	0.0788	0.0042
**Rye bread**	0.0486	0.0363	0.0654	0.0441	0.0151
